# Potential years of life lost due to oropharyngeal cancer in Brazil: 1979 to 2013

**DOI:** 10.11606/s1518-8787.2019053001054

**Published:** 2019-08-23

**Authors:** Lillia Magali Estrada Perea, Alexandra Crispim Boing, Marco Aurélio Peres, Antonio Fernando Boing

**Affiliations:** I Universidade Federal de Santa Catarina. Programa de Pós-Graduação em Saúde Coletiva. Florianópolis, SC, Brasil; II Universidade Federal de Santa Catarina, Departamento de Saúde Pública. Florianópolis, SC, Brasil; III Griffith University. Menzies Health Institute Queensland and School of Dentistry and Oral Health. Gold Coast, Australia

**Keywords:** Mouth Neoplasms, mortality, Pharyngeal Neoplasms, mortality, Potential Years of Life Lost, Time Series Studies

## Abstract

**OBJECTIVE:**

To estimate the years of life lost by the Brazilian population due to mouth and pharynx cancer from 1979 to 2013, and analyze the temporal trends in the studied period, according to the country’s region, sex and anatomical site.

**METHODS:**

The death records were obtained from the Mortality Information System and the data referring to the population, from the censuses of the Brazilian Institute of Geography and Statistics of 1980, 1991, 2000, 2010, and from intercensal estimates for the other years. The rates of potential years of life lost were calculated by applying the method suggested by Romeder and McWhinnie, and their trends were calculated using the Prais-Winsten method with first-order autocorrelation. The historical series were smoothed with the centered moving average technique of third order for white noise reduction.

**RESULTS:**

In the period from 1979 to 2013 in Brazil, there were a total of 107,506 premature deaths due to mouth and pharynx cancer, which generated a total of 1,589,501 potential years of life lost, the equivalent to a rate of 3.6 per 10,000 inhabitants. Males, whose rate was six times higher than for females, contributed with 85% of the years lost. The trends in the rates of years of life lost showed an annual 0.72% increase for men, 1.13% for women and 1.05% for pharynx cancer.

**CONCLUSIONS:**

The rate of potential years of life lost due to mouth and pharynx cancer in the country showed an upward trend within the studied period for both sexes, as well as for pharynx cancer and for the North, Northeast and Midwest regions.

## INTRODUCTION

Mouth and pharynx cancer are considered a public health problem all over the world. It is estimated that in 2015, 571,386 new cases of the disease emerged, and 316,168 people died as a result of these tumors worldwide. According to the International Agency for Research on Cancer, these values are expected to rise in the quinquennium from 2016 to 2020, reaching 350,000 deaths and 639,171 new cases in 2020^1^ .

In Latin America, the estimated number of deaths in 2015 was 33,925, with Brazil being the country with the largest number of cases in the region^1^ . The mortality rate for mouth and pharynx cancer in Brazil is 5.91/100,000 inhabitants, while other countries in the region, such as Argentina, Chile, Colombia and Mexico, did not have more than three deaths per 100,000 inhabitants^1^ . From 1979 to 2002, the mortality rate was 2.7 per 100,000 inhabitants^2^ , but in 2013, according to data from the Informatics Department of the Unified Health System (Datasus), the rate reached 3.6 deaths for each 100,000 inhabitants, indicating a 33% increase compared to the previous year.

The National Cancer Institute (Inca) estimates the incidence risk to be 7.8 per 100,000 inhabitants, with 11.27 new cases for every 100,000 men and 4.21 for every 100,000 women^3^ . It is estimated that in 2020, mouth and pharynx cancer will correspond to more than 21 thousand new cases and kill more than 10 thousand people in Brazil^1^ .

Although mortality rates allow the analysis of the risk of deaths in different regions of the world, they do not include these deaths’ impact on the communities, since they do not consider the age of death (early or otherwise). The potential years of life lost (PYLL) indicator covers premature mortality, the magnitude of causes and the social impact of diseases, since prematurity affects the individual’s economic and social potential in society. Moreover, this indicator is an alternative for the development of cancer prevention actions, enabling interventions in specific risk groups and assisting the improvement in the use of existing resources^4^ . The temporal trend analysis enhances the understanding of the dynamics, follow-up, and evolution of the deaths that occurred over the years, also allowing to make predictions, which contributes effectively to the planning of actions^5^ .

A search conducted in the PubMed database (Medline) in March 2018 on *“oral cancer”* and “ *potential years of life lost* ” did not identify recent studies analyzing the rates of years of life lost due to mouth and pharynx cancer in Brazil. Given this knowledge gap and the relevance of the theme, the present study was carried out to analyze the trend of premature mortality due to mouth and pharynx cancer in Brazil from 1979 to 2013.

## METHODS

Data on the deaths by mouth and pharynx cancer in Brazil of individuals aged between zero and 69 years old that occurred in the country from 1979, year of the beginning of data collection on the national level by the Ministry of Health’s Mortality Information System (SIM), until 2013, were analyzed. The observations ignoring sex, 26 in total (0.02% of the cases), were not included in the analysis of the rates according to sex.

The method for calculating PYLL is an adaptation of the proposal of Romeder and McWhinnie^6^ . It consists in the sum of the number of deaths in each age group multiplied by the number of years missing until the reference age, which was 69 years old in the present study. There are different cutoff points to define this reference age in the literature. The authors argue that, by including the deaths of people with higher ages, the weight given to it in the calculation of natural causes of death would be too great, and premature mortality would not be properly identified.

The deaths that occurred between 1979 and 1995 were classified according to the ninth revision of the International Classification of Diseases (ICD), and those that occurred between 1996 and 2013, according to the tenth revision. To allow the comparability of data throughout the period according to the main cause of death, codes 140.0 to 145.9 of ICD-9 and C00.0 to C8.9 of ICD-10 were classified as deaths by mouth cancer, and codes 146.0 to 149.9 of ICD-9 and C09 to C14.8 of ICD-10 as deaths by pharynx cancer, to neutralize the influence that the change in the coding could have on the results. The deaths were categorized according to the macro-region of residence, sex and age group (every five years).

The values of the resident population required for the calculation of the rates were obtained from the censuses performed by the Brazilian Institute of Geography and Statistics (IBGE) in 1980, 1991, 2000, 2010, and from intercensal estimates for the other years, available on the Datasus website.

The criteria for establishing the reference age used in the calculation of the potential years of life lost varies from author to author. A broad discussion regarding the inclusion or exclusion of deaths of children under one year old and the age to be considered as the upper limit can be found in the literature^4^ . Considering that the analysis of the present study includes only mouth and pharynx cancer and that the mortality due to this disease of children under one year is also of interest to the authors, the 50 deaths in this age group were not excluded (0.04% of the cases).

The original method for calculating PYLL uses Equation 1, where *d*_*i*_ represents the number of deaths between ages *i* and *i* + 1, and *a*_*i*_ represents the number of missing years until the reference age when death occurs between ages *i* and *i* + 1.

$$\sum_{i=0}^{69}a_{i}d_{i\;\;\;(1)}$$

The calculation of PYLL may be simplified by using five-year groups, as shown in Equation 2, with less than 2% divergence in the results^6^ .

$$\sum_{i=0}^{69}A_{q}D_{q\;\;\;(2)}$$

In this case, *D*_*q*_ represents the number of deaths in each quinquennium *q* , and *A*_*q*_ represents the difference between the quinquennium’s mean age *q* and the established reference age.

To compare PYLL between sexes, anatomical site and regions, the PYLL rates^7^ per 10,000 inhabitants were calculated, and subsequently standardized using the direct method of Equation 3. The Brazilian population of 2012 was used as the standard population.

$$\sum_{q=0}^{14}A_{q}\left(\frac{D_{q}}{P_{q}}\right)\;*\;\left(\frac{P_{qr}}{N_{r}}\right)*10.000\;\;(3)$$

In this equation, *P*_*q*_ = number of people whose ages are included in quinquennium *q* in the current population; *P*_*qr*_ = number of people whose ages are included in quinquennium *q* in the reference population; and *N*_*r*_ = number of people aged between zero and 69 years old in the reference population.

Finally, the temporal trend of PYLL rates was calculated according to the Prais-Winsten method with correction for first-order autocorrelation, according to equations 4 and 5:

$$–1\;+\;10^{b}\;=\;\Delta \;\;\;(4)$$

$$\Delta_{95\%\mathrm{CI}}\;=\;–\;1\;+\;10^{\left(b\;\pm \;t\;*\;se\right)}\;\;\;(5)$$

where “ *b* ” corresponds to the annual growth rate, “ *se* ” is the default error’s value, and the value of “ *t* ” is provided by Student’s t distribution. For further details on this methodology, the reader may consult the work developed by Antunes and Waldman^8^ .

The upward, downward or stagnation trend was expressed as annual percentage variation (APV) and was considered stationary when the regression coefficient did not differ from zero (p > 0.05). All analyses were carried out using version 13 of the Stata program ( *Stata Corp* ., *College Station* , USA).

To facilitate the visualization of the trend of the historical series in the graphs, it was smoothed with the centered moving average technique of third order for white noise reduction^9^ .

## RESULTS

During the study period, 146,925 deaths due to mouth and pharynx cancer were recorded, and 73.2% of them were of people under 70 years of age. Between 1979 and 2013, these deaths resulted in a total loss of 1,589,501 potential years of life in Brazil. In population terms, this value reflects an average rate of 3.6 PYLL per 10,000 inhabitants (data not shown).

There was a significant disparity in the distribution of premature deaths between men and women. Of the total years of life lost, 1,342,939 were attributed to males, with approximately eight out of 10 deaths having been premature. The ratio of the PYLL rate per 10,000 inhabitants between men and women was 6:1. The number of deaths and years of life lost with their rates, according to sex, may be seen in Table 1 . It is worth noting that this table does not present the 26 cases ignoring sex, which accounted for 0.02% of the sample and were disregarded in the calculations of the rates by sex.

**Table 1:** Number of deaths of people aged 0–69 years old, number of potential years of life lost (PYLL) and rate of life years lost (per 10,000 inhabitants) due to mouth and pharynx cancer, according to sex. Brazil, 1979–2013.

Year	Male			Female			Total		Rate
		
	Deaths	PYLL	Rate	Deaths	PYLL	Rate	Deaths	PYLL	
1979	1,329	21,576.0	5.85	208	2,999.5	0.76	1,537	24,575.5	3.28
1980	1,379	21,416.5	5.69	226	3,148.5	0.78	1,605	24,565.0	3.21
1981	1,381	21,812.0	5.67	276	4,165.0	1.00	1,657	25,977.0	3.31
1982	1,496	22,944.0	5.85	255	4,212.0	0.95	1,751	27,156.0	3.37
1983	1,479	22,361.5	5.52	247	3,827.0	0.86	1,726	26,188.5	3.16
1984	1,495	22,576.0	5.41	273	4,406.5	0.93	1,768	26,982.5	3.13
1985	1,599	23,712.0	5.59	253	3,967.5	0.85	1,852	27,679.5	3.18
1986	1,673	25,222.0	5.79	301	4,527.5	0.95	1,974	29,749.5	3.32
1987	1,759	26,985.5	5.94	286	4,339.5	0.85	2,045	31,325.0	3.34
1988	1,901	27,802.5	6.05	341	5,682.0	1.06	2,242	33,484.5	3.50
1989	1,956	29,040.0	6.13	343	5,676.5	1.03	2,299	34,716.5	3.52
1990	1,918	28,093.0	5.74	344	5,199.5	0.96	2,262	33,292.5	3.29
1991	2,064	29,928.0	5.95	340	5,529.5	0.98	2,404	35,457.5	3.40
1992	2,214	33,263.5	6.40	357	5,741.5	0.98	2,571	39,005.0	3.61
1993	1,562	22,554.0	4.22	265	4,572.5	0.75	1,827	27,126.5	2.43
1994	2,299	33,137.0	6.08	409	6,718.0	1.06	2,708	39,855.0	3.49
1995	2,339	34,162.0	6.03	422	6,299.0	0.99	2,761	40,461.0	3.43
1996	2,523	37,175.5	6.59	444	7,110.0	1.13	2,967	44,285.5	3.77
1997	2,569	38,352.5	6.69	419	6,556.0	1.02	2,988	44,908.5	3.76
1998	2,677	40,339.0	6.95	461	7,414.5	1.15	3,138	47,753.5	3.95
1999	2,833	41,942.5	7.20	484	8,008.5	1.21	3,317	49,951.0	4.10
2000	2,929	43,715.0	6.78	501	7,317.0	1.02	3,430	51,032.0	3.79
2001	2,959	44,511.5	6.82	532	8,400.0	1.16	3,491	52,911.5	3.88
2002	3,167	47,197.5	7.15	548	7,879.5	1.09	3,715	55,077.0	4.00
2003	3,426	51,500.0	7.71	532	8,225.0	1.10	3,958	59,725.0	4.28
2004	3,400	49,059.0	7.28	579	8,782.5	1.18	3,979	57,841.5	4.11
2005	3,585	52,895.5	7.63	612	9,549.5	1.24	4,197	62,445.0	4.31
2006	3,575	52,912.5	7.54	637	9,906.0	1.26	4,212	62,818.5	4.28
2007	3,682	54,342.0	6.79	631	9,616.5	1.09	4,313	63,958.5	3.81
2008	3,718	52,819.0	6.50	712	10,704.0	1.20	4,430	63,523.0	3.72
2009	3,956	56,078.0	6.72	745	10,607.5	1.16	4,701	66,685.5	3.81
2010	4,081	57,776.0	6.65	686	10,439.0	1.11	4,767	68,215.0	3.75
2011	4,143	58,380.0	6.67	779	12,002.0	1.26	4,922	70,382.0	3.84
2012	4,137	58,007.0	6.57	793	12,226.0	1.27	4,930	70,233.0	3.86
2013	4,311	59,351.0	6.33	751	10,806.5	1.07	5,062	70,157.5	3.56

In the analysis according to anatomical site, 46.3% of the premature deaths corresponded to mouth cancer, with an average PYLL rate of 1.7 per 10,000 inhabitants, thus contributing with 719,238 potential years of life lost. Pharynx cancer caused 53.7% of the premature deaths, contributing with 846,136 PYLL. The ratio between the PYLL rate per 10,000 inhabitants between pharynx and mouth cancer was 1.2:1, i.e., the PYLL rate for pharynx cancer was 20% higher than the one for mouth cancer. The number of deaths and years of life lost with their rates, according to anatomical site, may be seen in Table 2 . It is worth noting that this table presents the total number of deaths, including those ignoring sex, because the information of interest is the anatomical site.

**Table 2:** Number of deaths of people aged 0–69 years old, number of potential years of life lost (PYLL) and rate of life years lost (per 10,000 inhabitants) due to mouth and pharynx cancer, according to anatomical site. Brazil, 1979–2013.

Year	Mouth			Pharynx			Total		
		
	Deaths	PYLL	Rate	Deaths	PYLL	Rate	Deaths	PYLL	Rate
1979	819	12,736.5	1.72	718	11,839.0	1.56	1,537	24,575.5	3.28
1980	861	13,031.0	1.72	745	11,556.5	1.50	1,605	24,575.0	3.22
1981	882	13,509.5	1.74	776	12,485.0	1.58	1,658	25,994.5	3.31
1982	907	13,937.5	1.75	844	13,218.5	1.61	1,751	27,156.0	3.37
1983	898	13,315.0	1.63	828	12,873.5	1.53	1,726	26,188.5	3.16
1984	895	13,551.0	1.58	873	13,431.5	1.55	1,768	26,982.5	3.13
1985	931	13,812.0	1.60	921	13,867.5	1.58	1,852	27,679.5	3.18
1986	931	14,157.0	1.59	1.043	15,592.5	1.74	1,974	29,749.5	3.32
1987	990	14,909.5	1.60	1,056	16,428.0	1.74	2,046	31,337.5	3.35
1988	1,009	15,432.0	1.61	1,234	18,065.0	1.89	2,243	33,497.0	3.50
1989	1,062	15,724.0	1.61	1,237	18,992.5	1.91	2,299	34,716.5	3.52
1990	1,068	15,773.5	1.56	1,195	17,521.5	1.73	2,263	33,295.0	3.29
1991	1,127	16,631.0	1.60	1,279	18,831.5	1.80	2,406	35,462.5	3.40
1992	1,178	17,515.0	1.65	1,395	21,540.0	1.97	2,573	39,055.0	3.62
1993	796	12,084.0	1.08	1,034	15,149.5	1.35	1,830	27,233.5	2.44
1994	1,305	19,230.5	1.69	1,407	20,659.5	1.81	2,712	39,890.0	3.50
1995	1,267	18,022.0	1.54	1,497	22,521.0	1.89	2,764	40,543.0	3.43
1996	1,333	20,147.5	1.73	1,634	24,138.0	2.04	2,967	44,285.5	3.77
1997	1,384	20,748.5	1.74	1,608	24,225.0	2.02	2,992	44,973.5	3.77
1998	1,395	21,046.0	1.75	1,744	26,710.0	2.20	3,139	47,756.0	3.95
1999	1,489	22,201.5	1.83	1,829	27,782.0	2.27	3,318	49,983.5	4.10
2000	1,517	22,096.5	1.65	1,914	28,938.0	2.14	3,431	51,034.5	3.79
2001	1,607	24,457.0	1.80	1,884	28,454.5	2.08	3,491	52,911.5	3.88
2002	1,645	23,732.5	1.73	2,070	31,344.5	2.27	3,715	55,077.0	4.00
2003	1,810	26,970.0	1.94	2,148	32,755.0	2.34	3,958	59,725.0	4.28
2004	1,778	26,254.5	1.87	2,201	31,587.0	2.24	3,979	57,841.5	4.11
2005	1,904	28,093.0	1.94	2,293	34,352.0	2.37	4,197	62,445.0	4.31
2006	1,861	27,582.0	1.89	2,351	35,236.5	2.39	4,212	62,818.5	4.28
2007	2,036	29,962.0	1.79	2,277	33,996.5	2.03	4,313	63,958.5	3.81
2008	2,054	29,108.5	1.71	2,376	34,414.5	2.02	4,430	63,523.0	3.72
2009	2,182	30,768.5	1.76	2,519	35,917.0	2.05	4,701	66,685.5	3.81
2010	2,099	29,771.5	1.64	2,668	38,443.5	2.11	4,767	68,215.0	3.75
2011	2,219	30,861.5	1.68	2,703	39,520.5	2.16	4,922	70,382.0	3.84
2012	2,295	31,971.0	1.73	2,635	38,262.0	2.07	4,930	70,233.0	3.80
2013	2,385	32,831.5	1.66	2,677	37,326.0	1.89	5,062	70,157.5	3.56

The region of the country with the highest number of years of life lost was the Southeast (56.6%). The Southern region corresponded to 19.7% of the total PYLL, the Northeast region to 15.6%, the Midwest region to 5.6% and the North region showed the lowest rates, 2.5%. The Southeast and South regions had the highest PYLL rates, with values of 4.53 per 10,000 inhabitants and 4.48 per 10,000 inhabitants, respectively. The lowest rate was observed in the North region, with 1.50 per 10,000 inhabitants.

**Figure 1:**
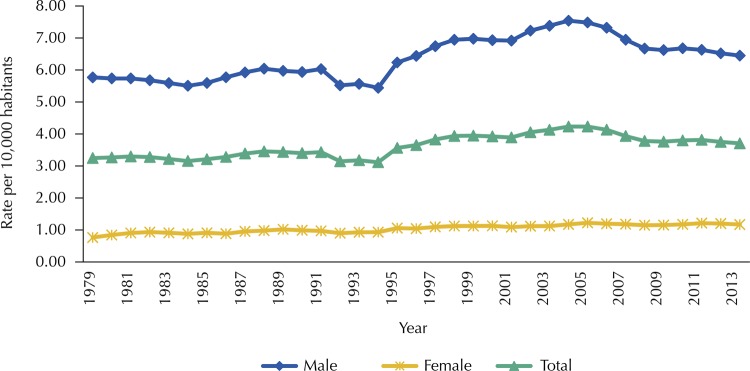
Historical series of the rates of potential years of life lost* due to mouth and pharynx cancer, according to sex. Brazil, 1979–2013.

The historical series of PYLL rates for mouth and pharynx cancer (together) showed an upward trend for both sexes. For men, APV was 0.72%, while for women, whose APV was 1.13%, the upward trend was more accentuated, as may be seen in Figure 1 .

* Rates standardized by the 2012 Brazilian population, with smoothing by moving averages of third order.

**Figure 2:**
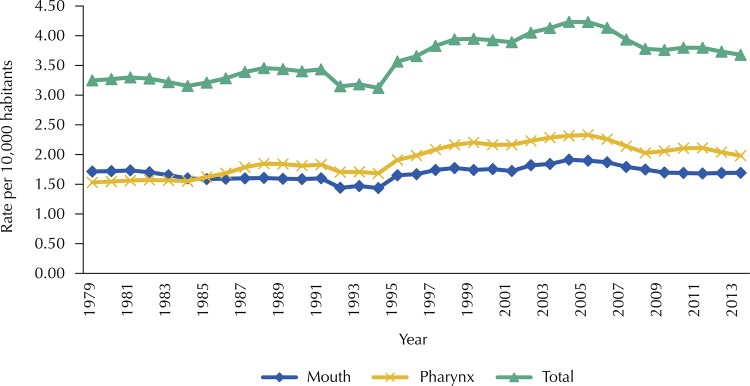
Historical series of the rates of potential years of life lost* due to mouth and pharynx cancer, according to anatomical site. Brazil, 1979–2013.

The historical series of APV rates for pharynx cancer also showed an upward trend, with 1.05% APV, and may be seen in Figure 2 . The premature mortality due to mouth cancer showed stability.

* Rates standardized by the 2012 Brazilian population, with smoothing by moving averages of third order.

**Figure 3:**
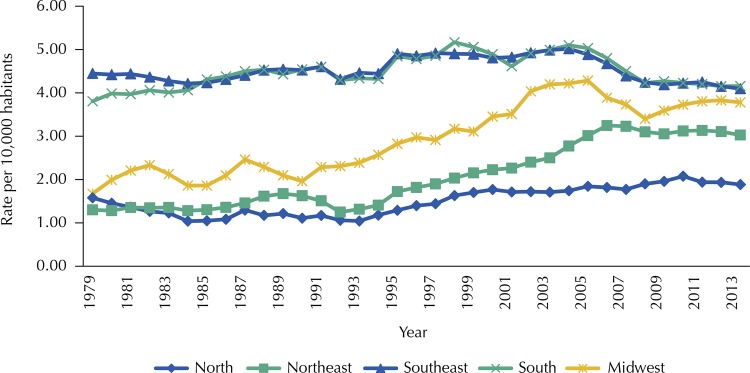
Historical series of the rates of potential years of life lost* due to mouth and pharynx cancer, according to region. Brazil, 1979–2013.

In the analysis by regions, the upward trend was observed for the Northern (APV: 1.63%), Midwest (APV: 2.63%) and Northeast regions (APV: 3.23%), which may be seen in Figure 3 and Table 3 . In the Southeast and South regions, the trends of premature mortality due to mouth and pharynx cancer showed stability. The Prais-Winsten regression coefficients and the PYLL rates’ APV according to region, sex and anatomical site are presented in Table 3 .

* Rates standardized by the 2012 Brazilian population, with smoothing by moving averages of third order.

**Table 3:** Coefficient and errors obtained in the Prais-Winsten regression, annual percentage variation, p-value and 95% confidence interval of the trend analysis for the rates of potential years of life lost due to mouth and pharynx cancer. Brazil, 1979–2013.

Variable	Regression coefficient	Standard error	p	Annual percentage variation	95%CI
Region					
North	0.0070377	0.0018149	0.000	1.63	0.70–2.57
Northeast	0.0138159	0.0017684	0.000	3.23	2.31–4.16
Southeast	-0.0000539	0.0008691	0.951	-0.01	-0.45–0.43
South	0.0012696	0.0009869	0.207	0.29	-0.21–0.79
Midwest	0.0112856	0.0015774	0.000	2.63	1.81–3.46
Site					
Mouth	0.0011698	0.0008179	0.162	0.27	-0.14–0.69
Pharynx	0.0045502	0.0010925	0.000	1.05	0.49–1.61
Sex					
Male	0.0030967	0.0009166	0.002	0.72	0.25–1.18
Female	0.0048772	0.0005882	0.000	1.13	0.83–1.43
Overall	0.0030144	0.0008302	0.001	0.71	0.32–1.11

## DISCUSSION

Premature mortality due to mouth and pharynx cancer showed an upward trend in the period between 1979 and 2013 in Brazil. Differences in intensity were identified according to sex and region of residence, with higher increase in females and in the Northeast region. The disease’s load analysis aims to describe as accurately as possible its impact on the lives of those who have it, and can be measured by several indicators, one of them being the potential years of life lost.

By incorporating trend analysis into the historical series of rates to study the behavior of premature mortality in the long term, the present study observed a positive annual percentage variation for both sexes in the rate of years of life lost due to mouth and pharynx cancer. According to the results observed by Alves and Neto, this reality is not very different from those of other neoplasms, which showed an increase in the premature mortality trend analysis for the period from 2000 to 2011^10^ .

The behavior of premature mortality rates for mouth and pharynx cancer in the long term is mainly affected by exposure in the past to the main risk factors, alcoholism and smoking. Studies show that adolescents start smoking increasingly early, especially males^11^ . It may be noted that the prevalence of the regular use of dental services was directly proportional to age, that is, the younger the patient, the lower the number of dentist appointments.^12^ In this study, the high number of years of life lost by the male population during the study period (85% of the total), as well as the PYLL rates’ ratio, according to which the value observed in men is 6 times higher than that observed in women, show a great loss in the young and productive male population. Even still, the trend analysis showed an increase in both sexes, with the annual percentage variation for women being twice that of men. The adequate use of health services, among other factors, such as early diagnosis, strongly influences premature mortality.

The worldwide prevalence of cigarette consumption almost tripled in the period between 1950 and 2009, but began to decline in the 1980s. In Brazil, a reduction in the prevalence of cigarette consumption among adults may be observed, with the proportion of men who use cigarettes being more than twice higher than that of women^13^ . Cigarette consumption is one of the main risk factors of mouth and pharynx cancer. It is expected that the changes in the prevalence of consumption are related to the behavior of the mortality rates of these types of cancer.

The anatomical site analysis shows that although the rates of mouth and pharynx cancer do not differ significantly, the PYLL rate was 20% higher for pharynx cancer than for mouth cancer. In addition, a 1% increase in the PYLL trend was observed for pharynx cancer during the study period. These findings suggest either greater difficulty in the timely diagnosis of pharynx cancer or an increase in the number of cases in this region, strongly associated with HPV infection.

The mortality rates of mouth and pharynx cancer have always been higher in the Southeast and South regions when compared to other regions^10^ . This could be related to the fact that the Southeast and South regions are associated with greater exposure to risk factors such as alcoholism and smoking^14^ . According to data from Datasus, the PYLL rate in the South region was 4.7 per 100,000 inhabitants in 2013, three times higher than the rate of the North region and two times higher than that of the Northeast region.

Considering that the PYLL rate is affected by both the age and the number of deaths, a high PYLL value was expected in the Southeast and South regions. These regions, despite having stable trends, showed an average PYLL rate that was three times higher than that of the North region, which had the lowest rate of all.

An increase in the trend of the PYLL rates was observed in the regions with the lowest mortality rates (North, Northeast and Midwest). The highest increase was observed in the Northeast region. The difference in the rate trends between regions obeys several conditions that influence inequality. The North and Northeast regions have the lowest rates of health professionals in activity^15^ . Due to its relationship with socioeconomic inequalities, mouth and pharynx cancer have a high mortality burden, especially in the most socio-economically vulnerable population groups.

The upward global trend of mouth and pharynx cancer found in this study would seem very close to stability if it was not masking the differences between the sexes. It is important to consider that this variation covers each year of a fairly long period, which means we cannot expect a linear behavior of these outcomes.

Due to the unavailability of similar studies in Brazil, it was not possible to compare the findings with previous studies. However, a study developed by Ibayashi et al.^16^ in Japan showed that, in 2005, men corresponded to 73% of the PYLL rate for mouth and pharynx cancer. These results are similar to those observed in the present study, which shows the impact of premature mortality among males. Ibayashi et al.^16^ observed that the PYLL rate in men (3.16 per 100,000) was four times higher than in women (0.78 per 100,000 inhabitants), having concluded that the specific sites responsible for the higher PYLL rate were oropharynx and tongue cancer, although they regretted not having used this indicator to analyze the rate for mouth and pharynx cancer more broadly, on both a national and international scale.

A limitation of this article refers to the interpretation of the rate trends, especially on the regional level, which requires caution, since regional inequalities in the collection of mortality data and the quality of the information systems should be considered. Analyses that are based on secondary records depend on the quality of the system of notification of deaths. The underreporting of deaths and the lack of accuracy in the definition of the cause of death may influence the results of the study by generating reduced rates that do not reflect reality. Similarly, the decrease in underreporting in regions characterized by poor quality of medical care may be influencing the increase in trends more than the increase in premature mortality itself.

As a positive aspect, it worth noting that the quality of the mortality data provided by SIM has been verified in several studies^17 , 18^ . These studies have observed a gradual increase in the system’s scope since its decentralization in 1992, and corroborate the adequate filling of the data in a percentage around 90%.

Health information systems in Brazil have shown great advances in the coverage of records. In the case of SIM, 96.1% coverage had already been reached in 2011. In the Southeast, South and Midwest regions, this coverage is close to 100%, while in the North and Northeast regions, only four federative units have coverage between 80% and 90%, and the others have coverage above 90%. With regard to the percentage of deaths with ill-defined underlying cause, a decline has been observed over the years, from 7.2% to 6.7% in the period from 2009 to 2011. The South and Midwest regions are those with lowest underreporting rates, with percentages of deaths due to ill-defined basic causes corresponding to 4.5% and 4.4%, respectively^19^ .

The reduction in deaths due to ill-defined causes, especially in the North and Northeast regions, as a result of the program of Reduction in the Percentage of Deaths due to Ill-defined Causes developed by the Ministry of Health’s Health Surveillance Secretariat, should be emphasized^20^ . The ratio of deaths due to ill-defined causes decreased from 45.7% to 23.7% in the period from 1979 to 2004, reaching 17.0% in 2005 and 8.0% in 2009^21^ .

Another limitation of the study could be the use of 69 years of age as the reference age, which may seem like an arbitrary choice in a study analyzing a population with discretionary life expectancy between genders. However, the reference age in this work is the age used in the methodology for calculation of the PYLL rate proposed by Romeder and McWhinnie^7^ , which considers premature the deaths of those under 70 years old. The use of the life expectancy according to gender is more appropriate when one wants to know the years of life lost during the productive period of each sex (WYPLL)^5^ . This method favors the analysis of premature deaths focusing on differentiated life expectancy and the retirement age of each sex to analyze the loss of productivity due to these deaths.

The results of this work emphasize the importance of studies analyzing the behavior of chronic diseases for subsequent development of prevention strategies. The high rates of years of life lost due to cancer, as well as to other chronic diseases, translate into a large volume of labor disabilities, burden on health systems and economic impact for families and society. All this may lead to social impairment and impoverishment^10^ .

The definition and calculation of the PYLL rate imply that higher rates correspond to a greater number of premature deaths. This ends up negatively impacting the economy, since premature deaths would affect the production of the young and productive population – not to mention the attempts of treatment before death, which generate high costs for the health system^22 , 23^ .

It is suggested that further studies on the trends of the PYLL rates are carried out to monitor the situation. This is the only way to learn more about the behavior of this disease and subsidize early detection strategies, timely diagnosis and treatment.
