# Supplementary material for: Potential years of life lost due to oropharyngeal cancer in Brazil: 1979 to 2013
Source: Rev Saude Publica. 2019 Aug 23;53:67. doi: 10.11606/s1518-8787.2019053001054 (PMC6707621; doi:10.11606/s1518-8787.2019053001054)
Supplement: Translation

# Translation: Anos potenciais de vida perdidos por câncer de boca e faringe no Brasil: 1979 a 2013

**Authors:** Lillia Magali Estrada Perea, Alexandra Crispim Boing, Marco Aurélio Peres, Antonio Fernando Boing

**Affiliations:** I Universidade Federal de Santa Catarina. Programa de Pós-Graduação em Saúde Coletiva. Florianópolis, SC, Brasil; II Universidade Federal de Santa Catarina, Departamento de Saúde Pública. Florianópolis, SC, Brasil; III Griffith University. Menzies Health Institute Queensland and School of Dentistry and Oral Health. Gold Coast, Australia

**Keywords:** Neoplasias Bucais, mortalidade, Neoplasias Faríngeas, mortalidade, Anos Potenciais de Vida Perdidos, Estudos de Séries Temporais

## Abstract

**OBJETIVO:**

Estimar os anos de vida perdidos pela população brasileira devido ao câncer de boca e faringe de 1979 a 2013 e analisar a tendências temporais no período estudado, segundo região do país, sexo e sítio anatômico.

**MÉTODOS:**

Os registros de óbitos foram obtidos do Sistema de Informações sobre Mortalidade e os dados referentes à população, dos censos do Instituto Brasileiro de Geografia e Estatística de 1980, 1991, 2000, 2010 e de estimativas intercensitárias para os demais anos. Foram calculadas as taxas de anos de vida potencialmente perdidos aplicando o método sugerido por Romeder e McWhinnie, e suas tendências foram calculadas usando o método de Prais-Winsten com correção para autocorrelação de primeira ordem. As séries históricas foram alisadas com técnica de média móvel central de ordem 3 para redução de ruído branco.

**RESULTADOS:**

No período de 1979 a 2013 no Brasil, houve um total de 107.506 mortes prematuras devido ao câncer de boca e faringe, o que gerou um total de 1.589.501 anos potenciais de vida perdidos, equivalendo a uma taxa de 3,6 por 10.000 habitantes. O sexo masculino, com uma taxa seis vezes maior que a do sexo feminino, contribuiu com 85% dos anos perdidos. As tendências das taxas de anos de vida perdidos mostraram aumento anual de 0,72% nos homens, 1,13% nas mulheres e 1,05% para o câncer de faringe.

**CONCLUSÕES:**

A taxa de anos potenciais de vida perdidos por câncer de boca e faringe no país mostrou tendência de aumento dentro do período estudado em ambos os sexos, assim como para o câncer de faringe e para as regiões Norte, Nordeste e Centro-Oeste.

## INTRODUÇÃO

O câncer de boca e de faringe é considerado um problema de saúde pública em todo o mundo. Estima-se que em 2015 tenham surgido 571.386 casos novos da doença e 316.168 pessoas tenham morrido em decorrência desses tumores em todo o mundo. Segundo a Agência Internacional de Pesquisa em Câncer, espera-se que tais valores apresentem ascensão no quinquênio de 2016 a 2020, chegando a 350.000 óbitos e 639.171 casos novos em 2020^1^ .

Na América Latina, o número estimado de mortes em 2015 foi de 33.925, sendo o Brasil o país com o maior número de casos na região^1^ . A taxa de mortalidade por câncer de boca e faringe no Brasil é de 5,91/100.000 habitantes, enquanto outros países da região, tais como Argentina, Chile, Colômbia e México, não superaram três óbitos por 100.000 habitantes^1^ . De 1979 a 2002, a taxa de mortalidade foi de 2,7 por 100.000 habitantes^2^ , mas em 2013, segundo dados do Departamento de Informática do Sistema Único de Saúde (Datasus), a taxa chegou a 3,6 óbitos para cada 100.000 habitantes, indicando um aumento de 33% em relação ao ano anterior.

O Instituto Nacional de Câncer (Inca) estima um risco de incidência de 7,8 por 100 mil habitantes, sendo 11,27 casos novos a cada 100 mil homens e 4,21 a cada 100 mil mulheres^3^ . Estima-se que no ano 2020 o câncer de boca e faringe apresente mais de 21 mil casos novos e mate mais de 10 mil pessoas no Brasil^1^ .

Ainda que as taxas de mortalidade permitam a análise do risco de óbitos nas diferentes regiões do mundo, elas não incluem o impacto das mortes nas comunidades, uma vez que não consideram a idade do óbito (precoce ou não). O indicador anos potenciais de vida perdidos (APVP) apresenta a mortalidade prematura, a magnitude das causas e o impacto social das doenças, uma vez que a prematuridade afeta o potencial econômico e social do indivíduo na sociedade. Além disso, esse indicador é uma alternativa para o desenvolvimento das ações preventivas para o câncer, possibilitando intervenções em grupos específicos de risco e auxiliando no aprimoramento do uso dos recursos já existentes^4^ . A análise de tendência temporal potencializa a o entendimento da dinâmica, do monitoramento, da evolução das mortes que ocorreram ao longo dos anos, inclusive possibilitando fazer previsões, o que contribui de maneira efetiva para o planejamento de ações^5^ .

Uma busca realizada na base de dados da PubMed (Medline) em março de 2018 sobre “ *oral cancer* ” e “ *potential years of life lost* ” não identificou estudos recentes analisando as taxas de anos de vida perdidos por câncer de boca e faringe no Brasil. Diante dessa lacuna de conhecimento e da relevância do tema, realizou-se o presente estudo para analisar a tendência da mortalidade prematura de câncer de boca e faringe no Brasil, no período de 1979 a 2013.

## MÉTODOS

Foram analisados os óbitos por câncer de boca e faringe no Brasil de indivíduos entre zero e 69 anos que ocorreram no país desde 1979, ano do início da coleta de dados em nível nacional pelo Sistema de Informações sobre Mortalidade (SIM) do Ministério da Saúde, até o ano 2013. As observações com sexo ignorado, 26 no total (0,02% dos casos), não foram incluídas na análise das taxas segundo sexo.

O método de cálculo dos APVP é uma adaptação da proposta de Romeder e McWhinnie^6^ . Ele consiste na soma do número de mortes em cada faixa etária multiplicado pelo número de anos faltantes para a idade-limite, que foi de 69 anos no presente estudo. Existem diferentes pontos de corte para definir essa idade-limite na literatura. Os autores argumentam que, ao se incluir as mortes de pessoas com idades maiores, seria dado um grande peso no cálculo às causas naturais de morte, e a mortalidade prematura não seria devidamente identificada.

Os óbitos ocorridos entre 1979 e 1995 foram classificados segundo a nona revisão da Classificação Internacional de Doenças (CID), e os ocorridos entre 1996 e 2013, segundo a décima revisão. Para permitir a comparabilidade dos dados de todo o período segundo a causa principal de morte, os códigos 140.0 a 145.9 da CID-9 e C00.0 a C8.9 da CID-10 foram classificados como óbitos por câncer de boca, e os códigos 146.0 a 149.9 da CID-9 e C09 a C14.8 da CID-10 como óbitos por câncer de faringe, no intuito de neutralizar a influência que a mudança da codificação poderia ter nos resultados. Os óbitos foram categorizados segundo macrorregião de residência, sexo e faixa etária (a cada cinco anos).

Os valores da população residente necessários para o cálculo das taxas foram obtidos por meio dos censos realizados pelo Instituto Brasileiro de Geografia e Estatística (IBGE) de 1980, 1991, 2000, 2010 e de estimativas intercensitárias para os demais anos, disponíveis no site do Datasus.

Os critérios para estabelecer a idade-limite usada no cálculo dos anos potencias de vida perdidos varia de autor para autor. Uma ampla discussão com respeito à inclusão ou exclusão das mortes em menores de um ano e sobre a idade a ser considerada como limite superior pode ser encontrada na literatura^4^ . Considerando que a análise do presente estudo inclui apenas o câncer de boca e faringe e que a mortalidade devido a essa doença em menores de um ano também é de interesse dos autores, não foram excluídas as 50 mortes nessa faixa etária (0,04% dos casos).

O método original para o cálculo dos APVP utiliza a equação 1, onde *d*_*i*_ representa o número de mortes entre as idades *i* e *i* + 1, e *a*_*i*_ representa o número de anos faltantes para a idade limite quando a morte ocorre entre as idades *i* e *i* + 1.

$$\sum_{i=0}^{69}a_{i}d_{i\;\;\;(1)}$$

Usando grupos de cinco anos, o cálculo do APVP pode ser simplificado, conforme mostrado na equação 2, com divergência de menos de 2% nos resultados^6^ .

$$\sum_{i=0}^{69}A_{q}D_{q\;\;\;(2)}$$

Nesse caso, *D*_*q*_ representa o número de mortes em cada quinquênio *q* , e *A*_*q*_ representa a diferença entre a idade média do quinquênio *q* e a idade limite estabelecida.

A fim de comparar os APVP entre sexos, sítio anatômico e regiões, foram calculadas a taxas de APVP^7^ por 10.000 habitantes, posteriormente padronizadas pelo método direto da equação 3. A população brasileira de 2012 foi utilizada como população-padrão.

$$\sum_{q=0}^{14}A_{q}\left(\frac{D_{q}}{P_{q}}\right)\;*\;\left(\frac{P_{qr}}{N_{r}}\right)*10.000\;\;(3)$$

Nessa equação, *P*_*q*_ = número de pessoas cujas idades estão compreendidas no quinquênio *q* na população atual; *P*_*qr*_ = número de pessoas cujas idades estão compreendidas no quinquênio *q* na população de referência; e *N*_*r*_ = número de pessoas com idade entre zero e 69 na população de referência.

Por fim, foi calculada a tendência temporal das taxas de APVP pelo método de Prais-Winsten com correção para autocorrelação de primeira ordem, conforme as equações 4 e 5:

$$–1\;+\;10^{b}\;=\;\Delta \;\;\;(4)$$

$$\Delta_{95\%\mathrm{CI}}\;=\;–\;1\;+\;10^{\left(b\;\pm \;t\;*\;se\right)}\;\;\;(5)$$

onde “ *b* ” corresponde à taxa de crescimento anual, “ *se* ” é o valor do erro padrão, e o valor de “ *t* ” é fornecido pela tabela da distribuição t de Student. Para maiores detalhes sobre essa metodologia, o leitor pode consultar o trabalho desenvolvido por Antunes e Waldman^8^ .

A tendência de aumento, declínio ou estagnação foi expressa como variação percentual anual (VPA) e foi considerada estacionária quando o coeficiente de regressão não diferiu de zero (p > 0,05). As análises foram realizadas no programa Stata, versão 13 ( *Stata Corp* ., *College Station* , EUA).

Para facilitar a visualização da tendência da série histórica nos gráficos, foi realizado seu alisamento pela técnica das médias móveis centrais de ordem 3 para redução de ruído branco^9^ .

## RESULTADOS

No período de estudo, foram registradas 146.925 mortes devido ao câncer de boca e faringe, sendo 73,2% delas de pessoas com menos de 70 anos de idade. Entre 1979 e 2013, um total de 1.589.501 anos potenciais de vida foi perdido no Brasil por essas mortes. Em termos populacionais, esse valor reflete uma taxa média de 3,6 APVP por 10.000 habitantes (dados não apresentados).

Observou-se expressiva disparidade na distribuição das mortes prematuras entre homens e mulheres. Do total de anos de vida perdidos, 1.342.939 foram atribuídos ao sexo masculino, com aproximadamente oito de cada 10 óbitos prematuros. A razão entre a taxa de APVP por 10.000 habitantes entre homens e mulheres foi de 6:1. O número de mortes e de anos de vida perdidos com as suas taxas, segundo sexo, pode ser observado na Tabela 1 . Vale salientar que essa tabela não apresenta os 26 casos com sexo ignorado, os quais representavam 0,02% da amostra e foram desconsiderados nos cálculos das taxas por sexo.

**Tabela 1:** Número de mortes de pessoas de 0–69 anos, número de anos potenciais de vida perdidos (APVP) e taxa de anos de vida perdidos (por 10.000 habitantes) por câncer de boca e faringe, segundo sexo. Brasil, 1979–2013.

Ano	Masculino			Feminino			Total		Taxa
		
	Mortes	APVP	Taxa	Mortes	APVP	Taxa	Mortes	APVP	
1979	1.329	21.576,0	5,85	208	2.999,5	0,76	1.537	24.575,5	3,28
1980	1.379	21.416,5	5,69	226	3.148,5	0,78	1.605	24.565,0	3,21
1981	1.381	21.812,0	5,67	276	4.165,0	1,00	1.657	25.977,0	3,31
1982	1.496	22.944,0	5,85	255	4.212,0	0,95	1.751	27.156,0	3,37
1983	1.479	22.361,5	5,52	247	3.827,0	0,86	1.726	26.188,5	3,16
1984	1.495	22.576,0	5,41	273	4.406,5	0,93	1.768	26.982,5	3,13
1985	1.599	23.712,0	5,59	253	3.967,5	0,85	1.852	27.679,5	3,18
1986	1.673	25.222,0	5,79	301	4.527,5	0,95	1.974	29.749,5	3,32
1987	1.759	26.985,5	5,94	286	4.339,5	0,85	2.045	31.325,0	3,34
1988	1.901	27.802,5	6,05	341	5.682,0	1,06	2.242	33.484,5	3,50
1989	1.956	29.040,0	6,13	343	5.676,5	1,03	2.299	34.716,5	3,52
1990	1.918	28.093,0	5,74	344	5.199,5	0,96	2.262	33.292,5	3,29
1991	2.064	29.928,0	5,95	340	5.529,5	0,98	2.404	35.457,5	3,40
1992	2.214	33.263,5	6,40	357	5.741,5	0,98	2.571	39.005,0	3,61
1993	1.562	22.554,0	4,22	265	4.572,5	0,75	1.827	27.126,5	2,43
1994	2.299	33.137,0	6,08	409	6.718,0	1,06	2.708	39.855,0	3,49
1995	2.339	34.162,0	6,03	422	6.299,0	0,99	2.761	40.461,0	3,43
1996	2.523	37.175,5	6,59	444	7.110,0	1,13	2.967	44.285,5	3,77
1997	2.569	38.352,5	6,69	419	6.556,0	1,02	2.988	44.908,5	3,76
1998	2.677	40.339,0	6,95	461	7.414,5	1,15	3.138	47.753,5	3,95
1999	2.833	41.942,5	7,20	484	8.008,5	1,21	3.317	49.951,0	4,10
2000	2.929	43.715,0	6,78	501	7.317,0	1,02	3.430	51.032,0	3,79
2001	2.959	44.511,5	6,82	532	8.400,0	1,16	3.491	52.911,5	3,88
2002	3.167	47.197,5	7,15	548	7.879,5	1,09	3.715	55.077,0	4,00
2003	3.426	51.500,0	7,71	532	8.225,0	1,10	3.958	59.725,0	4,28
2004	3.400	49.059,0	7,28	579	8.782,5	1,18	3.979	57.841,5	4,11
2005	3.585	52.895,5	7,63	612	9.549,5	1,24	4.197	62.445,0	4,31
2006	3.575	52.912,5	7,54	637	9.906,0	1,26	4.212	62.818,5	4,28
2007	3.682	54.342,0	6,79	631	9.616,5	1,09	4.313	63.958,5	3,81
2008	3.718	52.819,0	6,50	712	10.704,0	1,20	4.430	63.523,0	3,72
2009	3.956	56.078,0	6,72	745	10.607,5	1,16	4.701	66.685,5	3,81
2010	4.081	57.776,0	6,65	686	10.439,0	1,11	4.767	68.215,0	3,75
2011	4.143	58.380,0	6,67	779	12.002,0	1,26	4.922	70.382,0	3,84
2012	4.137	58.007,0	6,57	793	12.226,0	1,27	4.930	70.233,0	3,86
2013	4.311	59.351,0	6,33	751	10.806,5	1,07	5.062	70.157,5	3,56

Quando analisado o sítio anatômico, 46,3% das mortes prematuras corresponderam ao câncer de boca, apresentando uma taxa média de APVP de 1,7 por 10.000 habitantes e contribuindo, assim, com 719.238 anos potenciais de vida perdidos. Já o câncer de faringe ocasionou 53,7% das mortes prematuras, contribuindo com 846.136 APVP. A razão entre a taxa de APVP por 10.000 habitantes entre faringe e boca foi de 1,2:1, ou seja, a taxa de APVP por câncer de faringe foi 20% maior do que a taxa de APVP por câncer de boca. O número de mortes e de anos de vida perdidos com as suas taxas, segundo sítio anatômico, pode ser observado na Tabela 2 . Vale salientar que essa tabela apresenta o total de óbitos, incluídos aqueles com sexo ignorado, pois a informação de interesse é o sítio anatômico.

**Tabela 2:** Número de mortes de pessoas de 0–69 anos, número de anos potenciais de vida perdidos (APVP) e taxa de anos de vida perdidos (por 10.000 habitantes) por câncer de boca e faringe, segundo sítio anatômico. Brasil, 1979–2013.

Ano	Boca			Faringe			Total		
		
	Mortes	APVP	Taxa	Mortes	APVP	Taxa	Mortes	APVP	Taxa
1979	819	12.736,5	1,72	718	11.839,0	1,56	1.537	24.575,5	3,28
1980	861	13.031,0	1,72	745	11.556,5	1,50	1.605	24.575,0	3,22
1981	882	13.509,5	1,74	776	12.485,0	1,58	1.658	25.994,5	3,31
1982	907	13.937,5	1,75	844	13.218,5	1,61	1.751	27.156,0	3,37
1983	898	13.315,0	1,63	828	12.873,5	1,53	1.726	26.188,5	3,16
1984	895	13.551,0	1,58	873	13.431,5	1,55	1.768	26.982,5	3,13
1985	931	13.812,0	1,60	921	13.867,5	1,58	1.852	27.679,5	3,18
1986	931	14.157,0	1,59	1.043	15.592,5	1,74	1.974	29.749,5	3,32
1987	990	14.909,5	1,60	1.056	16.428,0	1,74	2.046	31.337,5	3,35
1988	1.009	15.432,0	1,61	1.234	18.065,0	1,89	2.243	33.497,0	3,50
1989	1.062	15.724,0	1,61	1.237	18.992,5	1,91	2.299	34.716,5	3,52
1990	1.068	15.773,5	1,56	1.195	17.521,5	1,73	2.263	33.295,0	3,29
1991	1.127	16.631,0	1,60	1.279	18.831,5	1,80	2.406	35.462,5	3,40
1992	1.178	17.515,0	1,65	1.395	21.540,0	1,97	2.573	39.055,0	3,62
1993	796	12.084,0	1,08	1.034	15.149,5	1,35	1.830	27.233,5	2,44
1994	1.305	19.230,5	1,69	1.407	20.659,5	1,81	2.712	39.890,0	3,50
1995	1.267	18.022,0	1,54	1.497	22.521,0	1,89	2.764	40.543,0	3,43
1996	1.333	20.147,5	1,73	1.634	24.138,0	2,04	2.967	44.285,5	3,77
1997	1.384	20.748,5	1,74	1.608	24.225,0	2,02	2.992	44.973,5	3,77
1998	1.395	21.046,0	1,75	1.744	26.710,0	2,20	3.139	47.756,0	3,95
1999	1.489	22.201,5	1,83	1.829	27.782,0	2,27	3.318	49.983,5	4,10
2000	1.517	22.096,5	1,65	1.914	28.938,0	2,14	3.431	51.034,5	3,79
2001	1.607	24.457,0	1,80	1.884	28.454,5	2,08	3.491	52.911,5	3,88
2002	1.645	23.732,5	1,73	2.070	31.344,5	2,27	3.715	55.077,0	4,00
2003	1.810	26.970,0	1,94	2.148	32.755,0	2,34	3.958	59.725,0	4,28
2004	1.778	26.254,5	1,87	2.201	31.587,0	2,24	3.979	57.841,5	4,11
2005	1.904	28.093,0	1,94	2.293	34.352,0	2,37	4.197	62.445,0	4,31
2006	1.861	27.582,0	1,89	2.351	35.236,5	2,39	4.212	62.818,5	4,28
2007	2.036	29.962,0	1,79	2.277	33.996,5	2,03	4.313	63.958,5	3,81
2008	2.054	29.108,5	1,71	2.376	34.414,5	2,02	4.430	63.523,0	3,72
2009	2.182	30.768,5	1,76	2.519	35.917,0	2,05	4.701	66.685,5	3,81
2010	2.099	29.771,5	1,64	2.668	38.443,5	2,11	4.767	68.215,0	3,75
2011	2.219	30.861,5	1,68	2.703	39.520,5	2,16	4.922	70.382,0	3,84
2012	2.295	31.971,0	1,73	2.635	38.262,0	2,07	4.930	70.233,0	3,80
2013	2.385	32.831,5	1,66	2.677	37.326,0	1,89	5.062	70.157,5	3,56

A região do país que apresentou o maior número de anos de vida perdidos foi a Sudeste (56,6%). A região Sul apresentou 19,7% dos APVP, a região Nordeste 15,6%, a região Centro-Oeste 5,6% e a região Norte apresentou o menor APVP, 2,5%. As regiões Sudeste e Sul tiveram as maiores taxas de APVP, com valores de 4,53 por 10.000 habitantes e 4,48 por 10.000 habitantes, respectivamente. A menor taxa foi observada na região Norte, com 1,50 por 10.000 habitantes.

A série histórica das taxas de APVP devido ao câncer de boca e faringe (conjuntamente) mostrou tendência ascendente em ambos os sexos. Nos homens a VPA foi de 0,72%, enquanto nas mulheres, cuja VPA foi de 1,13%, a tendência de aumento foi maior, como observado na Figura 1 .

**Figura 1:** Série histórica das taxas de anos potenciais de vida perdidos* devido ao câncer de boca e faringe, segundo sexo. Brasil, 1979–2013.

* Taxas padronizadas pela população brasileira de 2012, com alisamento por médias móveis de ordem 3.

A série histórica das taxas de APVP devido ao câncer de faringe também mostrou tendência de aumento, com VPA de 1,05%, e pode ser observada na Figura 2 . A mortalidade prematura devido ao câncer de boca apresentou estabilidade.

**Figura 2:** Série histórica das taxas de anos potenciais de vida perdidos* devido ao câncer de boca e faringe, segundo sítio anatômico. Brasil, 1979–2013.

* Taxas padronizadas pela população brasileira 2012, com alisamento por médias móveis de ordem 3.

Segundo a análise por regiões, a tendência de aumento nas taxas foi observada para as regiões Norte (VPA: 1,63%), Centro-Oeste (VPA: 2,63) e Nordeste (VPA: 3,23%), o que pode ser observado na Figura 3 e na Tabela 3 . Nas regiões Sudeste e Sul, as tendências da mortalidade prematura devido ao câncer de boca e faringe apresentaram estabilidade. Os coeficientes da regressão de Prais-Winsten, VPA das taxas de APVP por região, sexo e sítio anatômico podem ser observados na Tabela 3 .

**Figura 3:** Série histórica das taxas de anos potenciais de vida perdidos* devido ao câncer de boca e faringe, segundo região. Brasil, 1979–2013.

* Taxas padronizadas pela população brasileira 2012, com alisamento por médias móveis de ordem 3.

**Tabela 3:** Coeficiente e erros obtidos na regressão de Prais-Winsten, variação percentual anual, valor de p e intervalo de confiança de 95% da análise de tendência das taxas de anos potenciais de vida perdidos por câncer de boca e faringe. Brasil, 1979–2013.

Variável	Coeficiente da regressão	Erro-padrão	p	Variação percentual anual	IC95%
Região					
Norte	0,0070377	0,0018149	0,000	1,63	0,70–2,57
Nordeste	0,0138159	0,0017684	0,000	3,23	2,31–4,16
Sudeste	-0,0000539	0,0008691	0,951	-0,01	-0,45–0,43
Sul	0,0012696	0,0009869	0,207	0,29	-0,21–0,79
Centro-Oeste	0,0112856	0,0015774	0,000	2,63	1,81–3,46
Sítio					
Boca	0,0011698	0,0008179	0,162	0,27	-0,14–0,69
Faringe	0,0045502	0,0010925	0,000	1,05	0,49–1,61
Sexo					
Masculino	0,0030967	0,0009166	0,002	0,72	0,25–1,18
Feminino	0,0048772	0,0005882	0,000	1,13	0,83–1,43
Global	0,0030144	0,0008302	0,001	0,71	0,32–1,11

## DISCUSSÃO

A mortalidade prematura por câncer de boca e de faringe apresentou tendência de aumento no período entre 1979 e 2013 no Brasil. Foram identificadas diferenças de intensidade segundo sexo e região de residência, com maior ascensão no sexo feminino e na região Nordeste. A análise de carga da doença tem como objetivo descrever do modo mais preciso possível o impacto que determinada doença ou transtorno provoca na vida das pessoas quando as acomete e pode ser mensurada por vários indicadores, um deles sendo os anos potenciais de vida perdidos.

Ao incorporar a análise de tendências à série histórica das taxas para estudar o comportamento da mortalidade prematura em longo prazo, o presente trabalho observou variação percentual anual positiva para ambos os sexos na taxa de anos de vida perdidos devido ao câncer de boca e faringe. Segundo os resultados observados por Alves e Neto, essa realidade não é muito diferente do resto das neoplasias, que mostraram aumento na análise de tendência da mortalidade prematura abrangendo o período de 2000 a 2011^10^ .

O comportamento das taxas de mortalidade prematura por câncer de boca e faringe em longo prazo é afetado, principalmente, pela exposição no passado aos principais fatores de risco do câncer de boca e faringe, o etilismo e o tabagismo. Estudos mostram que adolescentes começam a fumar cada vez mais cedo, especialmente os do sexo masculino^11^ . Observa-se que a prevalência de uso regular de serviços odontológicos foi diretamente proporcional à idade, ou seja, quanto mais jovem, menor o número de consultas ao profissional da saúde^12^ . No presente trabalho, a grande quantidade de anos de vida perdidos pela população masculina durante o período de estudo (85% do total), assim como a razão das taxas de AVPV, na qual o valor observado nos homens é 6 vezes maior do que o observado nas mulheres, evidencia uma grande perda na população masculina jovem e produtiva. Ainda assim, a análise de tendência mostrou aumento em ambos os sexos, com a variação percentual anual nas mulheres sendo o dobro da dos homens. O uso adequado dos serviços de saúde, entre outros fatores, como o diagnóstico precoce, influencia fortemente a mortalidade prematura.

A prevalência mundial de consumo de cigarro quase triplicou no período entre 1950 e 2009, mas começou a declinar a partir de 1980. No Brasil, observa-se uma redução da prevalência do consumo de cigarros entre adultos, com uma proporção de homens que usam cigarros mais de duas vezes maior do que a de mulheres^13^ . O consumo de cigarro é um dos principais fatores de risco para o câncer de boca e faringe. Espera-se que as modificações na prevalência de consumo estejam relacionadas com o comportamento das taxas de mortalidade por esses tipos de câncer.

A análise segundo sítio anatômico mostra que, embora a razão de taxas entre boca e faringe não difira significativamente, a taxa de APVP por câncer de faringe foi 20% maior do que a taxa de APVP por câncer de boca. Além disso, foi observado aumento de 1% na tendência de APVP devido ao câncer de faringe durante o período estudado. Esses achados sugerem ou maior dificuldade no diagnóstico oportuno dos casos de câncer de faringe ou aumento dos casos de câncer nessa região, fortemente associado à infecção por HPV.

As taxas de mortalidade devido ao câncer de boca e faringe têm sido sempre maiores nas regiões Sudeste e Sul quando comparadas com as outras regiões^10^ . Isso poderia estar associado ao fato de as regiões Sudeste e Sul estarem associadas à maior exposição a fatores de risco como o etilismo e o tabagismo^14^ . Segundo os dados do Datasus, a taxa de APVP na região Sul para 2013 foi de 4,7 por 100.000 habitantes, três vezes maior que a taxa da região Norte e duas vezes maior do que a da região Nordeste.

Considerando que o AVPV é um indicador afetado tanto pela idade da morte quanto pelo número de mortes, era de se esperar um alto número de APVP nas regiões Sudeste e Sul. Essas regiões, embora tenham tendências estáveis, apresentaram uma taxa de APVP em média três vezes maior do que a região Norte, cuja taxa foi a menor de todas.

Foi observado aumento na tendência das taxas de APVP nas regiões com as menores taxas de mortalidade (Norte, Nordeste e Centro-Oeste). O maior aumento foi observado na região Nordeste. A diferença nas tendências das taxas entre regiões obedece a diversas condições que influenciam na desigualdade. As regiões Norte e Nordeste têm as menores taxas de profissionais de saúde em atividade^15^ . Devido a sua relação com as desigualdades socioeconômicas, o câncer de boca e faringe tem uma alta carga de mortalidade, especialmente nos grupos populacionais socioeconomicamente mais vulneráveis.

A tendência global ascendente de câncer de boca e faringe encontrada neste estudo poderia parecer uma variação bastante próxima da estabilidade se não estivesse mascarando as diferenças entre os sexos. É importante considerar que essa é uma variação para cada ano de um período bastante longo, no qual não podemos esperar comportamento linear de desfechos desse tipo.

Devido à indisponibilidade de estudos semelhantes no Brasil, não foi possível comparar os achados com estudos anteriores. Entretanto, estudo desenvolvido por Ibayashi et al.^16^ no Japão mostrou que, no ano de 2005, os homens apresentaram 73% dos APVP devido ao câncer de boca e faringe. Esses resultados são similares aos observados no presente estudo, no qual se evidencia o impacto da mortalidade prematura no sexo masculino. Ibayashi et al.^16^ observaram que a taxa de APVP nos homens (3,16 por 100.000) foi quatro vezes maior do que nas mulheres (0,78 por 100.000 habitantes), concluíram que os sítios específicos responsáveis pela maior quantidade de APVP eram orofaringe e língua e lamentaram que o câncer de boca e faringe não tenha sido mais amplamente analisado através deste indicador tanto em nível nacional como internacional.

Como limitação deste artigo, pode ser citada a interpretação das tendências das taxas, especialmente no nível regional, que requer cautela, uma vez que devem ser consideradas as desigualdades regionais no registro dos dados sobre mortalidade e a qualidade dos sistemas de informação. Análises realizadas com base nos registros secundários dependem da qualidade do sistema de notificação dos óbitos. O sub-registro de mortes e a falta de acurácia na definição da causa de morte podem influenciar os resultados do estudo ao gerar taxas reduzidas que não refletem a realidade. Da mesma forma, a diminuição da subnotificação em regiões caracterizadas por baixa qualidade da assistência médica pode estar influenciando no aumento das tendências mais do que um aumento na mortalidade prematura em si.

Como aspecto positivo, ressalta-se que a qualidade dos dados de mortalidade fornecidos pelo SIM tem sido verificada em vários estudos^17 , 18^ . Esses trabalhos têm observado o aumento gradativo da abrangência do sistema desde a sua descentralização em 1992 e corroboram o adequado preenchimento dos dados em uma porcentagem ao redor de 90%.

Os sistemas de informação em saúde no Brasil têm mostrado grande avanço na cobertura dos registros. No caso do SIM, já foi atingida uma cobertura de 96,1% em 2011. Nas regiões Sudeste, Sul e Centro-Oeste, essa cobertura é próxima de 100%, enquanto nas regiões Norte e Nordeste só quatro unidades federativas têm cobertura entre 80% e 90% e as demais têm cobertura acima de 90%. No que tange ao percentual de óbitos com causa básica mal definida, tem se observado uma queda através dos anos, passando de 7,2% para 6,7% no período de 2009 a 2011. As regiões Sul e Centro-Oeste são as que apresentam menor sub-registro, com percentuais de óbitos por causa básica mal definida de 4,5% e 4,4%, respectivamente^19^ .

Salienta-se a diminuição de óbitos por causas mal definidas, especialmente nas regiões Norte e Nordeste, como resultado do programa Redução do Percentual de Óbitos por Causas Mal Definidas desenvolvido pela Secretaria de Vigilância em Saúde do Ministério da Saúde^20^ . A proporção de mortes por causa mal definida passou de 45,7% a 23,7% no período de 1979 a 2004, chegando a 17,0% em 2005 e a 8,0% em 2009^21^ .

Poderia ser considerado também como limitação do estudo o uso de 69 anos como idade-limite, que pode parecer uma escolha arbitrária em um estudo analisando uma população com expectativas de vida discrepantes entre sexos. Entretanto, a idade considerada limite neste trabalho é a idade utilizada na metodologia de cálculo dos APVP proposta por Romeder e McWhinnie^7^ , que considera prematuras as mortes em menores de 70 anos. O uso da expectativa de vida de cada sexo é mais apropriado quando se quer conhecer os anos de vida perdidos durante o período produtivo de cada sexo (WYPLL)^5^ . Esse método favorece a análise das mortes prematuras com foco na expectativa de vida diferenciada e nas idades de aposentadoria de cada sexo para analisar a perda de produtividade devido a essas mortes.

Os resultados deste trabalho ressaltam a importância de estudos analisando o comportamento das doenças crônicas para posterior desenvolvimento de estratégias de prevenção. As altas taxas de anos de vida perdidos por câncer, assim como por outras doenças crônicas, se traduzem em um grande volume de incapacidades laborais, sobrecarga nos sistemas de saúde e impacto econômico para as famílias e para a sociedade. Tudo isso pode levar ao prejuízo social e empobrecimento^10^ .

A definição e o cálculo dos APVP implicam que maiores taxas correspondem a uma maior quantidade de mortes prematuras. Isso acaba impactando negativamente a economia, uma vez que as mortes prematuras afetariam a produção da população jovem e produtiva – sem contar as tentativas de tratamento antes da morte, que gera custos altos ao sistema de saúde^22 , 23^ .

Sugere-se que novos estudos sobre tendências das taxas de APVP sejam realizados, a fim de monitorar a situação. Só assim se poderá conhecer melhor o comportamento dessa doença e direcionar os recursos para estratégias de detecção precoce, assim como de diagnóstico e tratamento oportunos.
